# Mediating Role of Health Insurance on Socioeconomic Inequalities in Dental Utilisation Patterns Among Indonesian Adults

**DOI:** 10.1111/cdoe.70013

**Published:** 2025-08-24

**Authors:** Safira Khairinisa, Yusuke Matsuyama, Sakura Kiuchi, Diah Ayu Maharani, Jun Aida

**Affiliations:** ^1^ Department of Dental Public Health Graduate School of Medical and Dental Sciences, Institute of Science Tokyo Tokyo Japan; ^2^ Department of Preventive and Public Health Dentistry University of Indonesia Jakarta Indonesia; ^3^ Department of International and Community Oral Health Tohoku University Graduate School of Dentistry Sendai Japan; ^4^ Frontier Research Institute for Interdisciplinary Sciences, Tohoku University Sendai Japan

**Keywords:** dental insurance, dental service, health inequalities, mediation analysis, socioeconomic factors

## Abstract

**Objectives:**

From the perspective of Universal Health Coverage (UHC) to reduce health inequalities, health insurance plays a crucial role. This study examined the mediating effect of health insurance on the economic and educational inequalities in dental utilisation patterns among Indonesian adults.

**Methods:**

This cross‐sectional study analysed self‐reported data from participants (*n* = 26 351) of the Indonesian Family Life Survey‐5 (IFLS‐5) conducted during the transition of Indonesia's health financing system in 2014–2015. Economic and educational inequalities in dental utilisation were measured and examined using the relative concentration index (RCI). A multinomial logistic regression, adjusted for confounders (sex, age, ethnicity, religion, marital status, household size and residency based on province and rural–urban), examined the association of economic status (quintiles of adjusted monthly household expenditure) and educational status (unschooled to higher education) with dental utilisation patterns (never/irregular/regular). The extent to which the association was explained by health insurance ownership (public and private insurance) was assessed using the Karlson–Holm–Breen mediation method.

**Results:**

Among participants, 12.9% of individuals utilise dental visits irregularly and 1.4% regularly, with the utilisation concentrated among those with higher economic status (RCI: 0.30, standard error [SE]: 0.01) and educational status (RCI: 0.34, SE: 0.01). Compared to those with the lowest economic quintile, the highest economic quintile showed higher odds of irregular utilisation (odds ratio [OR]: 2.16; 95% confidence interval [CI]: 1.89–2.48) and regular utilisation (OR: 4.28; 95% CI: 2.50–7.34). People with higher education were more likely to utilise dental care, with higher odds ratios of irregular utilisation (OR: 6.80; 95% CI: 5.04–9.18) and regular utilisation (OR: 7.34; 95% CI: 2.24–24.04) compared to unschooled individuals. Private insurance partly mediated the association with regular dental utilisation: stronger indirect effects were observed at the highest economic level (proportion mediated [PM]: 10.6%) and highest educational level (PM: 9.2%). In contrast, the mediation effects of public insurance were less remarkable.

**Conclusion:**

Education and economic status play a significant role in determining dental utilisation patterns, with limited mediating effects for public insurance on these associations. To ensure equitable access to quality dental utilisation across socioeconomic groups, it is crucial to strengthen public insurance programmes that effectively address the needs of disadvantaged populations.

## 1 Introduction

Oral diseases represent a major global health and economic challenge, affecting the quality of life and contributing to significant disability and financial burden [1, 2]. In 2019, the global prevalence of oral diseases was estimated to have reached 44.5%, resulting in significant disability‐adjusted life years (DALYs) and an economic burden of approximately US$ 710 billion [3, 4]. The high cost of dental care can exacerbate social inequalities in access to dental care [2]. In response, the World Health Organisation (WHO) has emphasised the integration of dental care into Universal Health Coverage (UHC) to achieve universal oral health coverage by 2030 [1, 5].

However, health financing systems vary across countries, and their effectiveness may depend on the situation and social context of each country [6, 7]. While health insurance is widely seen as a mechanism to reduce inequalities, the persistent global burden of oral disease, particularly in low‐ and middle‐income countries where three‐quarters of affected individuals live, highlights the need to re‐evaluate and strengthen systematic approaches to improving access to dental care [8, 9].

Indonesia, the fourth most populated country, faces significant challenges in providing quality health care across its 6000 islands and decentralised healthcare system [10, 11]. Prior to 2014, the country's health insurance system was fragmented, consisting of separate schemes tailored to specific groups such as civil servants or low‐income populations. However, dental coverage within these schemes was inconsistent and sometimes left out [12]. To address these inequities, the government introduced the National Health Insurance (NHI) program (Jaminan Kesehatan Nasional, JKN) in January 2014, unifying the fragmented public insurance schemes into a single, comprehensive framework. JKN explicitly included basic dental services, such as examinations, consultations, emergency treatment, extractions, medications, fillings, and annual scaling, making them accessible to both government‐subsidised and self‐paying members [13, 14]. At the same time, private health insurance has remained a viable option, especially for higher income groups, typically offering more extensive and flexible benefits, but is unaffordable for the majority [11]. This coexistence of public and private insurance systems in Indonesia provides a unique context to examine how different health financing models mediate socioeconomic inequalities [11, 15].

Economic and educational inequalities have long been barriers to equitable access to health care, as higher education promotes awareness and prioritisation of health, while economic challenges limit access to both preventive and curative services [16, 17]. Health insurance has the potential to reduce these inequalities by reducing out‐of‐pocket expenses and improving access to care, but even after several years of JKN implementation, recent surveys in Indonesia have shown persistently low utilisation rates and high national dental caries prevalence, at 88% in 2018 and 82.8% in 2023 [18, 19, 20, 21]. This study hypothesises that different types of health insurance differentially mediate the association between economic and educational status and dental utilisation in Indonesia. Specifically, we expect that private insurance will mediate the association more strongly than public insurance. Therefore, this study aimed to quantitatively examine the mediating effects of public and private health insurance on economic and educational inequalities in dental utilisation using 2014–2015 national data, a period that provides a baseline for the current healthcare financing system.

## 2 Methods

This cross‐sectional observational study was reported according to the Strengthening the Reporting of Observational Studies in Epidemiology (STROBE) guidelines [22].

### 2.1 Data Source

The Indonesian Family Life Survey (IFLS) was a large‐scale longitudinal study conducted by RAND Corporation in 1993, with subsequent waves conducted in 1997, 2000, 2007, and 2014–2015. This study focused on the most recent available wave of the IFLS (IFLS‐5), conducted from September 2014 to March 2015, during the transition period of the health insurance system introduced in January 2014 [23, 24]. The survey used a multistage stratified sampling design that covered 83% of the Indonesian population in 13 of all 34 provinces, including all five Javanese provinces, four Sumatran provinces and four provinces from other major island groups (Bali, West Nusa Tenggara, South Kalimantan and South Sulawesi), which were selected based on survey cost while ensuring representation of socio‐economic and ethnic diversity [23, 25]. The household and individual response rates were 92% and 90.5%, respectively [23, 26]. From the published datasets, 31 420 records were available from the questionnaire for individuals aged 15 years and older who reported their utilisation of outpatient health care, including dental utilisation [23]. Individuals younger than 22 years old (*n* = 4858) and other incomplete cases of observed variables (*n* = 211) were excluded. As a result, the final complete case dataset consists of *n* = 26 351.

### 2.2 Variables

#### 2.2.1 Exposure Variables

This study used economic and educational status as exposure variables. In developing countries such as Indonesia, household consumption has a greater advantage in reflecting financial status than income as a long‐term household standard of living [25, 27]. This household‐level consumption measure includes food, non‐food consumables, durable goods, education, and housing. The combined household expenditure was divided by the adult equivalence scale, calculated using the formula: (*A* + α𝐾)^θ^, where *A* was the number of adults, *K* was the number of children, α was the cost of living for children (0.5 in developing countries), and *θ* indicated the economic scale of the family (0.75) [27, 28]. This monthly expenditure was categorised into quintiles: Q1 (lowest) to Q5 (highest) [29].

Respondents were also asked about their highest educational attainment, which was classified into five levels of education: unschooled, elementary school, junior high school, senior high school and higher education [10]. Only individuals aged 22 years or older were included in this study. This age threshold was chosen based on the assumption that by this age, individuals are likely to have completed the mandatory 12 years of education and, if applicable, higher education such as university [30]. Including younger individuals could lead to misclassification of final educational attainment, as some may report their current level of education while still in the process of completing their studies and potentially bias the comparison between groups.

#### 2.2.2 Outcome Variable

The outcome variable was dental utilisation, categorised based on responses to the IFLS‐5 question, ‘How regularly do you have your teeth checked?’ Participants were classified into three groups: ‘never,’ ‘irregular,’ and ‘regular’ based on original responses.

#### 2.2.3 Mediator Variable

Information on insurance ownership was obtained from self‐reported questionnaires, ‘Are you the policyholder/primary beneficiary of health benefits or health insurance?’ and ownership was recorded as ‘yes’ or ‘no’ [23, 31]. Those who answered ‘yes’ were further classified. In early 2014, JKN was launched, consolidating all major public health insurance schemes (Askes, Jamkesmas, Jamsostek and Jamkesda) under a single agency. These were categorised as public insurance in this analysis [32]. Other options, including employer‐provided medical reimbursement, employer‐provided clinics, private health insurance and savings‐related insurance, were categorised as private insurance in this study. In the multinomial logistic regression, health insurance was combined into a single variable of four categories: no ownership, private only, public only and dual coverage (both public and private insurance). This categorisation reflected the reality in Indonesia, where some individuals may have both public and private insurance. For the mediation analysis, private and public insurance were treated as separate two binary variables (0 = no insurance; 1 = has insurance). This approach was done to isolate their distinct mediating effects on the association between socioeconomic status and dental utilisation for each insurance type.

#### 2.2.4 Confounding Variables

The following confounders were assessed by self‐reported questionnaire and recategorised as follows: sex (male/female), age (reclassified as 22–34 years old, 35–44 years old, 45–64 years old and ≥ 65 years old), ethnicity (Javanese/non‐Javanese), religion (Muslim/non‐Muslim), marital status (currently married/not currently married), household size (1, 2–4 and > 4), and residency [25]. Residency was derived from province and rural–urban status variables, with provinces regrouped into Java and non‐Java based on their socioeconomic and cultural similarity [27]. Urban or rural areas were determined using the Indonesian Bureau of Statistics (BPS) classification [24, 25].

### 2.3 Statistical Analysis

Descriptive analyses were performed to summarise the characteristics of the study participants by cross‐tabulation. A concentration curve provides a comprehensive view of health inequalities by showing the proportion of dental utilisation attributable to the cumulative proportions of individuals ranked from poorest to richest and from lowest to highest educational status. The concentration index, calculated as twice the area between the concentration curve and the line of equality, assesses the degree of economic and educational inequality in dental utilisation [28]. However, because dental utilisation was treated as a binary variable (regular and irregular user vs. non‐user) when calculating the concentration index, the estimated concentration index does not necessarily fall within the range of −1 to 1. In line with Wagstaff's approach, the value was normalised as the relative concentration index (RCI) by dividing it by 1 − *μ*, where *μ* is the mean of dental utilisation [16, 28].

Univariable multinomial logistic regression was used to estimate the unadjusted odds ratios (ORs) and their 95% confidence intervals (CIs) for each dental utilisation pattern on exposures and mediators. Multivariable multinomial logistic regression was then used to calculate the fully adjusted ORs for each dental utilisation pattern, simultaneously adjusting for exposures, mediators and confounders. Results are presented as ORs with their corresponding 95% CIs for each utilisation pattern (irregular and regular).

**FIGURE 1:**
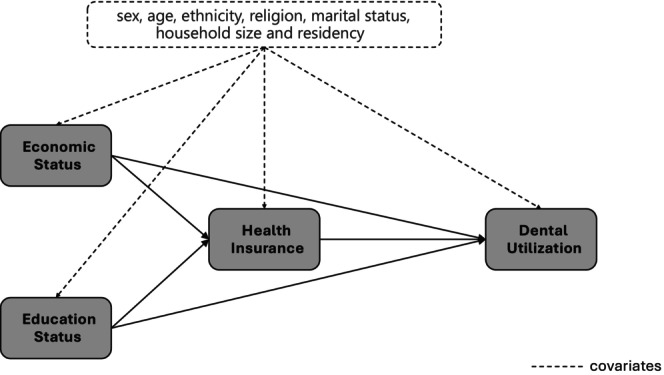
Directed acrylic graph for the mediation analysis.

Using the Karlson–Holm–Breen (KHB) method, a mediation analysis based on Figure 1 was conducted to calculate the total, direct and indirect effects, to estimate the role of health insurance (public and private insurance) in the association of each socioeconomic status (economic and education separately) with dental utilisation. Within the KHB framework, multinomial logistic regression analysis was applied to estimate ORs and 95% CIs. The analysis was performed using the khb package in Stata [33]. The proportion mediated (PM) quantified the extent to which the effect of an independent variable on an outcome was explained by a mediating variable (PM = *ß* indirect effect/*ß* total effect × 100) [34]. The contribution of public and private insurance as mediators was disentangled [33]. Model 1 was unadjusted and each exposure was separately included, while Model 2 was adjusted for all confounders. For the sensitivity analysis, multinomial logistic regression models with and without the inclusion of mediators (public and private insurance) were compared for each exposure to estimate the total, direct, and indirect effects (Supporting Information 1). The KHB analysis was also performed for each type of insurance separately (Supporting Information 2). All models were estimated by complete case analysis. The significance level was set at a *p*‐value < 0.05.

## 3 Results

**FIGURE 2:**
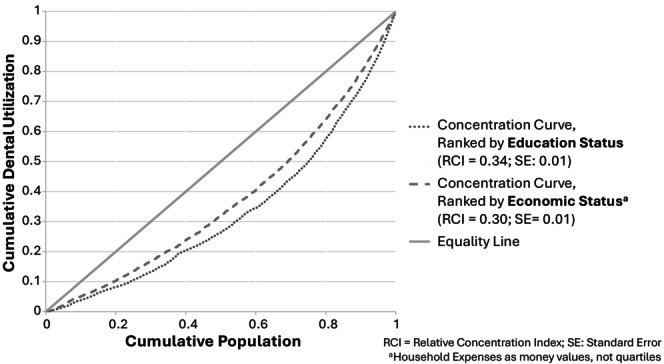
Relative concentration index (RCI) of economic and educational inequalities in dental utilisation.

Table 1 shows the sociodemographic characteristics associated with health insurance ownership among 26 351 adults. In the total sample, 50.4% (13 281 individuals) had no health insurance, while 49.6% (13 070 individuals) had some form of health insurance. Among those with health insurance, the majority, 46.6% (12 286 individuals) of the total sample, were covered by public insurance, while only 7.0% (1837 individuals) had private insurance. Figure 2 illustrates the distribution of dental utilisation across economic and educational levels. Dental utilisation was both pro‐rich and pro‐educated, as indicated by the concentration curve below the equity line, with RCI 0.30 (SE: 0.01) for economic and RCI 0.34 (SE: 0.01) for education status.

**TABLE 1:** Descriptive statistics by health insurance ownership (*n* = 26 351).

	Total		Health insurance ownership (any)				Public insurance		Private insurance	
			No		Yes		Yes		Yes	
Variable
*n*	%column	*n*	%row	*n*	%row	*n*	%row	*n*	%row
Total	26 351	100.0	13 281	50.4	13 070	49.6	12 286	46.6	1837	7.0
**Dental utilization**										
Never	22 583	85.7	11 909	52.7	10 674	47.3	10 161	45.0	1191	4.5
Irregularly	3403	12.9	1277	37.5	2126	62.5	1912	56.2	496	14.6
Regularly	365	1.4	95	26.0	270	74.0	213	58.4	150	41.1
**Education status**										
Unschooled	1228	4.7	745	60.7	483	39.3	481	39.2	2	0.2
Elementary school	8844	33.6	4952	56.0	3892	44.0	3823	43.2	135	1.5
Junior high school	4927	18.7	2721	55.2	2206	44.8	2133	43.3	219	4.4
Senior high school	7520	28.5	3548	47.2	3972	52.8	3643	48.4	819	10.9
Higher education	3832	14.5	1315	34.3	2517	65.7	2206	57.6	662	17.3
**Economic status**										
Q1 (Low)	5190	19.7	2742	52.8	2448	47.2	2421	46.6	77	1.5
Q2	5231	19.9	2788	53.3	2443	46.7	2372	45.3	184	3.5
Q3	5289	20.1	2859	54.1	2430	45.9	2329	44.0	291	5.5
Q4	5237	19.9	2572	49.1	2665	50.9	2474	47.2	454	8.7
Q5 (High)	5404	20.5	2320	42.9	3084	57.1	2690	49.8	831	15.4
**Age**										
22–34	10 365	39.3	5377	51.9	4988	48.1	4592	44.3	967	9.3
35–44	6865	26.1	3277	47.7	3588	52.3	3362	49.0	524	7.6
45–64	7465	28.3	3756	50.3	3709	49.7	3565	47.8	327	4.4
≥ 65	1656	6.3	871	52.6	785	47.4	767	46.3	19	1.1
**Marital status**										
Married	21 832	82.9	10 849	49.7	10 983	50.3	10 320	47.3	1527	7.0
Not married	4519	17.1	2432	53.8	2087	46.2	1966	43.5	310	6.9
**Sex**										
Female	14 075	53.4	7089	50.4	6986	49.6	6614	47.0	798	5.7
Male	12 276	46.6	6192	50.4	6084	49.6	5672	46.2	1039	8.5
**Residency**										
Java‐Urban	9525	36.1	4274	44.9	5251	55.1	4846	50.9	932	9.8
Java‐Rural	4851	18.4	2820	58.1	2031	41.9	1977	40.8	166	3.4
Non Java‐Urban	5876	22.3	2534	43.1	3342	56.9	3111	52.9	509	8.7
Non Java‐Rural	6099	23.1	3653	59.9	2446	40.1	2352	38.6	230	3.8
**Ethnicity**										
Javanese	11 685	44.3	6157	52.7	5528	47.3	5182	44.3	801	6.9
Non Javanese	14 666	55.7	7124	48.6	7542	51.4	7104	48.4	1036	7.1
**Religion**										
Muslim	23 642	89.7	12 116	51.2	11 526	48.8	10 870	46.0	1570	6.6
Non Muslim	2709	10.3	1165	43.0	1544	57.0	1416	52.3	267	9.9
**Household size**										
One	1855	7.0	1072	57.8	783	42.2	727	39.2	128	6.9
Two to four	21 607	82.0	10 772	49.9	10 835	50.1	10 185	47.1	1511	7.0
More than four	2889	11.0	1437	49.7	1452	50.3	1374	47.6	198	6.9

Table 2 shows the results of multinomial regression analyses, examining the associations between economic status, education and health insurance with different patterns of dental utilisation (irregular and regular). In the fully adjusted model, individuals in higher economic quintiles (Q2–Q5) had progressively higher odds of regular or irregular dental utilisation compared to those in the lowest quintile (Q1). For example, those in the highest quintile (Q5) had an OR of 2.16 (95% CI: 1.89–2.48) for irregular utilisation, while the OR was 4.28 (95% CI: 2.50–7.34) for regular utilisation. Similarly, individuals with higher education were more likely to utilise dental care, with higher odds of both irregular (OR: 6.80; 95% CI: 5.04–9.18) and regular utilisation (OR: 7.34; 95% CI: 2.24–24.04) compared to unschooled individuals.

**TABLE 2:** Odds ratios of economic, education, and health insurance for dental utilization by multinomial logistic regression (*n* = 26 351).

	Irregular utilisation (*n* = 3403; 12.9%)			Regular utilisation (*n* = 365; 1.4%)		
	*n*/prevalence of dental utilisation (%^a^)	Univariable OR (95% CI)	Multivariable OR^b^ (95% CI)	*n*/prevalence of dental utilisation (%^a^)	Univariable OR (95% CI)	Multivariable OR^b^ (95% CI)
**Health insurance (Ref: no insurance)**	1277 (9.6)	1.00	1.00	95 (0.7)	1.00	1.00
Public only	1628 (14.5)	1.60** (1.48–1.73)	1.32** (1.21–1.43)	120 (1.1)	1.59** (1.21–2.08)	1.25 (0.95–1.64)
Private only	212 (27.5)	3.95** (3.33–4.68)	1.92** (1.60–2.30)	57 (7.4)	14.26** (10.15–20.04)	5.05** (3.52–7.25)
Both	284 (26.6)	3.84** (3.30–4.46)	2.16** (1.84–2.54)	93 (8.7)	16.90** (12.57–22.72)	7.15** (5.22–9.80)
**Economic status (Ref: Q1)**	364 (7.0)	1.00	1.00	16 (0.3)	1.00	1.00
Q2	465 (8.9)	1.30** (1.13–1.50)	1.13 (0.98–1.31)	37 (0.7)	2.35** (1.31–4.23)	1.79 (0.99–3.23)
Q3	577 (10.9)	1.64** (1.43–1.88)	1.29** (1.12–1.49)	49 (0.9)	3.16** (1.79–5.56)	1.94* (1.09–3.45)
Q4	796 (15.2)	2.41** (2.12–2.75)	1.64** (1.43–1.88)	83 (1.6)	5.73** (3.35–9.79)	2.72** (1.57–4.72)
Q5	1201 (22.2)	3.94** (3.48–4.47)	2.16** (1.89–2.48)	180 (3.3)	13.45** (8.05–22.47)	4.28** (2.50–7.34)
**Highest educational attainment (Ref: unschooled)**	54 (4.4)	1.00	1.00	3 (0.2)	1.00	1.00
Elementary school	645 (7.3)	1.71** (1.29–2.28)	1.81** (1.36–2.42)	35 (0.4)	1.67 (0.51–5.45)	1.38 (0.42–4.57)
Junior high school	465 (9.4)	2.27** (1.70–3.03)	2.47** (1.83–3.34)	25 (0.5)	2.20 (0.66–7.30)	1.43 (0.42–4.88)
Senior high school	1160 (15.4)	4.04** (3.06–5.35)	3.70** (2.75–4.97)	137 (1.8)	8.59** (2.73–27.02)	3.58* (1.10–11.72)
Higher education	1079 (28.2)	9.04** (6.82–11.99)	6.80** (5.04–9.18)	165 (4.3)	24.89** (7.93–78.12)	7.34** (2.24–24.04)

Abbreviations: CI, confidence interval; OR, odd ratio.

^a^ Percentage represents the prevalence of people with irregular and regular utilisation within each group. This is calculated by dividing the total number of people with irregular or regular utilisation in each exposure group (e.g., insurance status, economic status and educational attainment) by the total population in that exposure group.

^b^ Multivariable model adjusted with confounders (sex, age, ethnicity, religion, marital status, household size, and residency).

* significant at *p* < 0.05

** significant at *p* < 0.01

Table 3 presents the results of the mediation analysis using KHB methods, illustrating how health insurance mediates the association of economic and educational factors with dental utilisation patterns. The indirect effects through public and private health insurance on the association of economic status with irregular dental utilisation were not significant for those with economic status Q2 and Q3, while they became greater and significant for those with Q4 (OR: 1.04; 95% CI: 1.02–1.06; total PM = 4.7%) and Q5 (OR: 1.09; 95% CI: 1.07–1.12; total PM = 7.2%). Most of the mediation between economic and irregular utilisation was through private insurance (PM in Q2 = 4.2%–Q5 = 7.1%) and not remarkable or statistically giving an opposing effect through public insurance.

**TABLE 3:** Mediation analysis between economic and educational status with dental utilization through health insurance (*n* = 26 351).

	Irregular utilisation		Regular utilisation	
	Model 1	Model 2	Model 1	Model 2
	OR (95% CI)	OR (95% CI)	OR (95% CI)	OR (95% CI)
**Economic status (Ref: Q1)**				
**Q2**				
Total effect	1.29** (1.12–1.49)	1.23** (1.06–1.42)	2.20** (1.22–3.97)	2.02** (1.12–3.65)
Direct effect	1.28** (1.11–1.48)	1.23** (1.06–1.42)	2.12** (1.18–3.82)	1.99** (1.10–3.60)
Indirect effect	1.01 (0.99–1.03)	1.00 (0.98–1.02)	1.04* (1.00–1.08)	1.01 (0.98–1.05)
%Total PM	4.3	0.1	4.7	2.0
%PM by public insurance	−2.1	−4.1	−0.7	−1.3
%PM by private insurance	6.3	4.2	5.4	3.2
**Q3**				
Total effect	1.62** (1.41–1.86)	1.53** (1.33–1.76)	2.79** (1.58–4.93)	2.52** (1.42–4.46)
Direct effect	1.59** (1.38–1.82)	1.52** (1.32–1.74)	2.60** (1.47–4.59)	2.42** (1.37–4.28)
Indirect effect	1.02** (1.00–1.04)	1.01 (0.99–1.03)	1.08** (1.03–1.12)	1.04** (1.01–1.08)
%Total PM	4.5	1.8	7.1	4.5
%PM by public insurance	−2.2	−0.1	−1.1	−1.6
%PM by private insurance	6.7	5.1	8.2	6.2
**Q4**				
Total effect	2.40** (2.11–2.74)	2.22** (1.94–2.54)	4.86** (2.83–8.34)	4.32** (2.51–7.44)
Direct effect	2.26** (1.98–2.58)	2.14** (1.87–2.45)	4.17** (2.43–7.16)	3.88** (2.25–6.70)
Indirect effect	1.06** (1.04–1.09)	1.04** (1.02–1.06)	1.17** (1.12–1.22)	1.11** (1.07–1.15)
%Total PM	6.9	4.7	9.4	7.3
%PM by public insurance	0.3	−0.7	0.2	−0.4
%PM by private insurance	6.6	5.4	9.5	7.7
**Q5**				
Total effect	3.96** (3.49–4.49)	3.53** (3.10–4.01)	10.93** (6.52–18.34)	10.31** (15.47–1.00)
Direct effect	3.49** (3.08–3.97)	3.23** (2.83–3.67)	8.06** (4.78–13.58)	7.23** (4.27–12.25)
Indirect effect	1.13** (1.10–1.16)	1.09** (1.07–1.12)	1.36** (1.29–1.43)	1.27** (1.21–1.32)
%Total PM	9.1	7.2	12.8	10.7
%PM by public insurance	0.9	0.1	0.6	0.1
%PM by private insurance	8.1	7.1	12.2	10.6
**Education status (Ref: unschooled)**				
**Elementary school**				
Total effect	1.71** (1.29–2.27)	1.92** (1.43–2.56)	1.60** (0.49–5.21)	1.50 (0.45–4.93)
Direct effect	1.67** (1.26–2.22)	1.90** (1.42–2.54)	1.54** (0.47–5.01)	1.49 (0.45–4.91)
Indirect effect	1.02 (1.00–1.05)	1.01 (0.99–1.03)	1.04* (1.00–1.08)	1.01 (0.97–1.04)
%Total PM	4.2	1.4	8.4	1.3
%PM by Public insurance	2.4	1.8	2.7	3.2
%PM by Private insurance	1.7	−0.4	5.7	−1.9
**Middle school**				
Total effect	2.25** (1.69–3.01)	2.79** (2.07–3.77)	1.93 (0.58–6.40)	1.76 (0.52–6.00)
Direct effect	2.16** (1.62–2.88)	2.72** (2.02–3.67)	1.75 (0.53–5.81)	1.69 (0.50–5.74)
Indirect effect	1.04** (1.02–1.07)	1.03** (1.00–1.05)	1.10** (1.06–1.14)	1.05** (1.01–1.09)
%Total PM	5.2	2.4	14.7	7.8
%PM by public insurance	1.6	1.4	2.0	2.9
%PM by private insurance	3.6	1.0	12.7	4.9
**High school**				
Total effect	4.00** (3.03–5.30)	4.67** (3.48–6.26)	6.87** (2.18–21.65)	5.52** (1.70–17.95)
Direct effect	3.61** (2.73–4.78)	4.35** (3.24–5.84)	5.42** (1.72–17.10)	4.72** (1.45–15.35)
Indirect effect	1.11** (1.08–1.14)	1.07** (1.05–1.10)	1.27** (1.21–1.33)	1.17** (1.12–1.22)
%Total PM	7.5	4.6	12.3	9.2
%PM by public insurance	2.2	1.5	1.5	1.5
%PM by private insurance	5.3	3.1	10.8	7.7
**Higher education**				
Total effect	9.10** (6.86–12.07)	10.46** (7.79–14.06)	19.99** (6.35–62.90)	15.57** (4.80–50.52)
Direct effect	7.63** (5.75–10.13)	9.12** (6.79–12.27)	13.52** (4.28–42.69)	11.50** (3.54–37.39)
Indirect effect	1.19** (1.15–1.23)	1.15** (1.11–1.18)	1.48** (1.38–1.58)	1.35** (1.27–1.44)
%Total PM	8.0	5.8	13.0	11.0
%PM by public insurance	2.7	2.0	1.9	1.8
%PM by private insurance	5.3	3.9	11.1	9.2

*Note:* Model 1 = Unadjusted model (Economic ***or*** Education status ➔ Health insurance (Public ***and*** private insurance) ➔ Dental utilization. Model 2 = Model 1 adjusted with confounders (sex, age, ethnicity, religion, marital status, household size, and residency)).

Abbreviations: CI, confidence interval; OR, odds ratio; PM, proportion mediated.

* Significant at *p* < 0.05.

** Significant at *p* < 0.01.

Similarly, as also reported in Table 3, the indirect effects of health insurance on irregular dental utilisation also increased with the level of education. The significant indirect effects compared to those unschooled were: middle school (OR: 1.03; 95% CI: 1.00–1.05; Total PM = 2.4%), high school (OR: 1.07; 95% CI: 1.05–1.10; Total PM = 4.6%) and higher education (OR: 1.15; 95% CI: 1.11–1.18; Total PM = 5.8%). The mediation between education and irregular utilisation was greater through public insurance for those who only finished elementary school (PM = 1.8%) and middle school (PM = 1.4%) but greater through private insurance for those who finished high school (PM = 3.1%) and higher education (PM = 3.9%).

For regular dental utilisation, more pronounced socioeconomic gradients than irregular dental utilisation were observed. The indirect effects of public and private health insurance increased across economic quantiles. Compared to Q1, the OR for Q2 was insignificant, but progressively stronger and significant associations were observed for Q3 (OR: 1.04; 95% CI: 1.01–1.08; Total PM = 4.5%), Q4 (OR: 1.11; 95% CI: 1.07–1.15; Total PM = 7.3%), and Q5 (OR: 1.27; 95% CI: 1.21–1.32; Total PM = 10.7%). Most of the mediation between economic and regular utilisation was through private insurance (PM in Q2 = 3.2%–Q5 = 10.6%) and not remarkable through public insurance.

Regarding the indirect effects of public and private insurance on the association of educational status with regular dental utilisation, compared to those unschooled, the indirect effects were insignificant for those in elementary school, but significant for those in middle school (OR: 1.05; 95% CI: 1.01–1.09; Total PM = 7.8%), high school (OR: 1.17; 95% CI: 1.12–1.22; Total PM = 9.2%) and higher education (OR: 1.35; 95% CI: 1.27–1.44; Total PM = 11.0%). The mediation between education and regular utilisation was greater through public insurance for those who only finished elementary school (PM = 3.2%), but greater through private insurance for those who finished middle school (PM = 4.9%), high school (PM = 7.7%) and higher education (PM = 9.2%) The additional analysis, having public and private health insurance separately, showed that the indirect effects and PM through public insurance were generally small and not significant (Table S2.1), whereas private insurance consistently showed significant associations (Table S2.2).

## 4 Discussion

The distributional patterns identified in this study showed economic and educational inequalities in dental utilisation across Indonesia. Individuals with higher economic and educational status were significantly more likely to have dental utilisation, with the inequality gaps being more pronounced for regular than irregular utilisation. Individuals with the highest economic quintile and education status showed the highest levels of utilisation, which was partly explained by greater access to private insurance. In contrast, public insurance contributed minimally, reflecting its limited variation across socioeconomic groups and possibly insufficient coverage of dental services. Overall, the role of health insurance varied across socioeconomic groups and was generally small.

This study has several limitations that should be considered. First, although the IFLS sampling frame covers approximately 83% of Indonesia's population, it focused predominantly on more developed provinces, where areas with lower utilisation rates may not be fully represented [14]. Second, the reliance on self‐reported data introduces the possibility of social desirability and recall bias. However, the anonymity provided to respondents may have mitigated social desirability effects and given the low overall utilisation rates, it is unlikely that participants misreported their dental care patterns, particularly as frequency was not directly assessed [23, 31]. Third, the absence of an explicit operational definition for ‘regular’ and ‘irregular’ dental utilisation in the national dataset presents a limitation, as respondents likely relied on subjective interpretations that potentially affected the consistency of the findings. It is also important to acknowledge that not all insured individuals utilise their dental coverage, and some might access dental services through other means, which could influence the observed associations.

Furthermore, the study's cross‐sectional design limits the ability to establish causal relationships, especially given the timing of data collection during the transitional period of health insurance implementation and its focus on lifetime dental utilisation patterns as the outcome [35]. We applied the KHB decomposition method to explore mediation effects [33], and although reverse causation is theoretically possible, we consider it unlikely in this setting. Because subsidised public plans and employer‐based coverage were available, immediate dental needs are less likely to drive people to have health insurance [11, 12]. Furthermore, while dental care plays an important role for overall health, it is often not the primary factor influencing health insurance enrollment [36, 37]. Moreover, our framework assumes that higher socioeconomic status and education lead to improved access to health insurance, which subsequently facilitates greater healthcare utilisation, consistent with existing evidence on the social determinants of health [15, 38, 39]. Finally, unmeasured factors, such as oral health problems and other confounding variables, may also have influenced the results.

The strength of this study is that the stratification of the social determinants allowed for the observation of progressive trends within each group, providing a detailed and comprehensive view of the interplay between the exposures, health insurance types and dental utilisation. This approach enriched the results by providing deeper insights into the gradients of social inequality and access to dental care across different socioeconomic strata. In addition, this study may provide a baseline statistic of the implementation of Indonesia's NHI law for comparison with future research, although these data may not reflect the current pattern due to changes in health insurance coverage, dental services and population behaviour since the time of data collection.

Health insurance plays a critical role in improving access to care by reducing financial barriers, increasing utilisation of services and promoting healthier behaviours [8]. In addition, having insurance may indirectly increase dental care utilisation by allowing individuals to reallocate savings from covered medical expenses [31, 40]. However, this study shows that despite its availability, health insurance provides limited mediation, with economic and educational status remaining key determinants of dental utilisation. In Indonesia, dental care utilisation has historically depended more on an individual's ability to pay, with wealthier groups accessing services more frequently despite greater needs among lower income groups [18, 19]. Ideally, public insurance should reduce the inequality in dental utilisation across economic or educational groups. The fact that public insurance made only marginal contributions, while private insurance disproportionately benefits more advantaged groups, suggests that the scheme may not adequately address the structural barriers faced by vulnerable populations.

The limited mediation effects of health insurance that additional factors, such as awareness, logistical barriers, service availability and individual perceptions of need and accessibility, must also be addressed in order to improve dental care utilisation across all populations [13, 40, 41, 42]. Low awareness of oral health leads many people to neglect dental care. Wealthier and more educated individuals have better health‐seeking behaviour and the ability to afford higher quality, tailored care [17, 35, 43, 44, 45]. However, even among those who prioritise oral health, they often face limited public insurance coverage or other structural barriers, forcing them to either forgo treatment or pay out of pocket, making dental care more accessible to individuals with higher economic and educational status [10, 37, 41, 46].

Despite the availability of private insurance, public insurance should remain mandatory for all to ensure access to basic healthcare [9, 11, 47]. However, while UHC aims for equal access, it can unintentionally deepen inequalities by benefiting advantaged groups more if interventions fail to address varying needs and barriers [48]. These gaps highlight the importance of moving beyond equal access to focus on health equity [45, 49]. Private insurance, while a practical option for those who can afford it, offers higher‐quality care and more comprehensive options, potentially easing financial strain on public systems [40, 43, 44]. Targeted interventions are crucial for addressing inequalities, including subsidising disadvantaged groups, strengthening health infrastructure, improving quality of care, increasing oral health literacy and focusing on systematic prevention as a cost‐effective effort to reduce the overall burden [11, 12, 45]. Discrepancies between policy and implementation further emphasise the need to measure health care coverage based on actual services received, considering barriers such as health worker absenteeism, drug shortages and patient adherence [50]. Regular monitoring of public insurance financing is essential to ensure a sustainable national health system that benefits all [51]. Future studies should use more recent data and broader indicators, including oral health status, literacy, reasons for non‐utilisation, dentist‐to‐population ratios and healthcare accessibility, to better understand how different insurance types mediate the utilisation of dental care across groups [13, 14, 27, 36].

## 5 Conclusion

Education and economic status are key factors influencing health insurance ownership and dental care utilisation, with higher socioeconomic groups more likely to have private insurance. Although public insurance covers half of the study population, its impact on dental utilisation remains limited, suggesting that expanding coverage alone is insufficient. To promote oral health equity, reforms must address structural barriers, subsidise vulnerable groups, enhance service quality and increase oral health awareness to ensure equitable access for all.

## Ethics Statement

IFLS‐5 was ethically approved by the University of Gadjah Mada Research Ethics Committee in Indonesia and the RAND Human Subjects Protection Committee (No s0064‐06‐01‐CR01). Additional ethics approval for this study was not required as this study was a secondary data analysis in which data are publicly available in a de‐identified format.

## Conflicts of Interest

The authors declare no conflicts of interest.

## Supporting information


**Tables S1–S2:** cdoe70013‐sup‐0001‐TableS1‐S2.docx.

## Data Availability

The data that support the findings of this study are available in RAND Indonesian Family Life Survey at https://www.rand.org/well‐being/social‐and‐behavioral‐policy/data/FLS/IFLS.html [23, 24]. These data were derived from the following resources available in the public domain: IFLS Public Use Data, https://www.rand.org/well‐being/social‐and‐behavioral‐policy/data/FLS/IFLS/access.html and are accessible via the Rand Labor and Population website (https://www.rand.org/labor/FLS/IFLS.html).

## References

[1] N. Jain , U. Dutt , I. Radenkov , and S. Jain , “WHO'S Global Oral Health Status Report 2022: Actions, Discussion, and Implementation,” Oral Diseases 30, no. 2 (2024): 73–79, 10.1111/odi.14516. 36680388

[2] C. R. Vernazza , S. Birch , and N. B. Pitts , “Reorienting Oral Health Services to Prevention: Economic Perspectives,” Journal of Dental Research 100, no. 6 (2021): 576–582, 10.1177/0022034520986794. 33478327 PMC8138330

[3] M. Jevdjevic and S. Listl , “Global, Regional, and Country‐Level Economic Impacts of Oral Conditions in 2019,” Journal of Dental Research 104, no. 1 (2024): 17–21, 10.1177/00220345241281698. 39535193 PMC11662506

[4] C. Tu , G. Wang , Z. Hu , S. Wang , Q. Yan , and X. Liu , “Burden of Oral Disorders, 1990–2019: Estimates From the Global Burden of Disease Study 2019,” Archives of Medical Science 19, no. 4 (2023): 930–940, 10.5114/aoms/165962. 37560733 PMC10408023

[5] M. A. Peres , L. M. D. Macpherson , R. J. Weyant , et al., “Oral Diseases: A Global Public Health Challenge,” Lancet 394, no. 10194 (2019): 249–260, 10.1016/S0140-6736(19)31146-8. 31327369

[6] B. Garla , G. Satish , and K. Divya , “Dental Insurance: A Systematic Review,” Journal of International Society of Preventive & Community Dentistry 4, no. 5 (2014): 73–77, 10.4103/2231-0762.146200. PMC4278106 25558454

[7] J. Aida , K. Fukai , and R. G. Watt , “Global Neglect of Dental Coverage in Universal Health Coverage Systems and Japan's Broad Coverage,” International Dental Journal 71, no. 6 (2021): 454–457, 10.1016/j.identj.2020.12.027. 33618834 PMC9275350

[8] B. Dong , “The Impact of Basic Health Insurance Participation Characteristics on the Health of Mobile Populations: The Mediating Role of Health Service Utilization Behavior,” Frontiers in Public Health 12 (2024): 1243703, 10.3389/fpubh.2024.1243703. 38362214 PMC10867968

[9] World Health Organization , “Global Oral Health Status Report: Towards Universal Health Coverage for Oral Health by 2030,” (2022), https://www.who.int/publications/i/item/9789240061484.

[10] A. D. Laksono , Z. K. Nantabah , R. D. Wulandari , A. Khoiri , and M. Tahangnacca , “Barriers to Expanding the National Health Insurance Membership in Indonesia: Who Should the Target?,” Journal of Primary Care & Community Health 13 (2022): e111112, 10.1177/21501319221111112. PMC9280783 35818670

[11] A. B. Pratiwi , H. Setiyaningsih , M. O. Kok , T. Hoekstra , A. G. Mukti , and E. Pisani , “Is Indonesia Achieving Universal Health Coverage? Secondary Analysis of National Data on Insurance Coverage, Health Spending and Service Availability,” BMJ Open 11, no. 10 (2021): e050565, 10.1136/bmjopen-2021-050565. PMC8491299 34607864

[12] R. Agustina , T. Dartanto , R. Sitompul , et al., “Universal Health Coverage in Indonesia: Concept, Progress, and Challenges,” Lancet 393, no. 10166 (2019): 75–102, 10.1016/S0140-6736(18)31647-7. 30579611

[13] I. Dewanto , S. Koontongkaew , and N. Widyanti , “Characteristics of Dental Services in Rural, Suburban, and Urban Areas Upon the Implementation of Indonesia National Health Insurance,” Frontiers in Public Health 8 (2020): 138, 10.3389/fpubh.2020.00138. 32478026 PMC7235165

[14] D. Erlangga , S. Ali , and K. Bloor , “The Impact of Public Health Insurance on Healthcare Utilization in Indonesia: Evidence From Panel Data,” International Journal of Public Health 64, no. 4 (2019): 603–613, 10.1007/s00038-019-01215-2. 30737522 PMC6517357

[15] D. A. Maharani and A. Rahardjo , “Is the Utilization of Dental Care Based on Need or Socioeconomic Status? A Study of Dental Care in Indonesia From 1999 to 2009,” International Dental Journal 62, no. 2 (2012): 90–94, 10.1111/j.1875-595X.2011.00095.x. 22420478 PMC9374974

[16] S. Rezaei , M. H. Pulok , T. Zahirian Moghadam , and H. Zandian , “Socioeconomic‐Related Inequalities in Dental Care Utilization in Northwestern Iran,” Clinical, Cosmetic and Investigational Dentistry 12 (2020): 181–189, 10.2147/CCIDE.S253242. 32425612 PMC7196241

[17] A. Ghanbarzadegan , P. Bastani , L. Luzzi , and D. Brennan , “Inequalities in Utilization and Provision of Dental Services: A Scoping Review,” Systematic Reviews 10, no. 1 (2021): 222, 10.1186/s13643-021-01779-2. 34376247 PMC8356458

[18] Indonesian Ministry of Health , “Basic Health Researches (RISKESDAS),” (2007), https://repository.badankebijakan.kemkes.go.id/id/eprint/4378/.

[19] Indonesian Ministry of Health , “Basic Health Researches (RISKESDAS),” (2013), https://repository.badankebijakan.kemkes.go.id/id/eprint/4467/.

[20] Indonesian Ministry of Health , “Basic Health Researches (RISKESDAS),” (2018), https://repository.badankebijakan.kemkes.go.id/id/eprint/3514/.

[21] Indonesian Ministry of Health , “Survey Kesehatan, Indonesia,” (2023).

[22] S. Cuschieri , “The STROBE Guidelines,” Saudi Journal of Anaesthesia 13, no. 5 (2019): 31, 10.4103/sja.SJA_543_18. PMC6398292 30930717

[23] RAND , “RAND IFLS‐5 Study Description,” accessed February 21, 2024, https://www.rand.org/well‐being/social‐and‐behavioral‐policy/data/FLS/IFLS/ifls5.html.

[24] RAND , “The Indonesian Family Life Survey,” accessed February 21, 2024, https://www.rand.org/well‐being/social‐and‐behavioral‐policy/data/FLS/IFLS.html.

[25] T. Aizawa , “Inequality in Health Opportunities in Indonesia: Long‐Term Influence of Early‐Life Circumstances on Health,” BMC Public Health 22 (2022): 1334, 10.1186/s12889-022-13714-8. 35831815 PMC9278321

[26] H. Holipah , H. W. Sulistomo , and A. Maharani , “Tobacco Smoking and Risk of all‐Cause Mortality in Indonesia,” PLoS One 15, no. 12 (2020): e0242558, 10.1371/journal.pone.0242558. 33259522 PMC7707492

[27] J. Mulyanto , D. S. Kringos , and A. E. Kunst , “The Evolution of Income‐Related Inequalities in Healthcare Utilisation in Indonesia, 1993–2014,” PLoS One 14, no. 6 (2019): e0218519, 10.1371/journal.pone.0218519. 31237901 PMC6592526

[28] O. O'Donnell , E. van Doorslaer , A. Wagstaff , and M. Lindelow , Analyzing Health Equity Using Household Survey Data (World Bank, 2007).

[29] M. Griselda , S. D. Alfian , I. A. Wicaksono , M. Wawruch , and R. Abdulah , “Findings From the Indonesian Family Life Survey on Patterns and Factors Associated With Multimorbidity,” Scientific Reports 13 (2023): 18607, 10.1038/s41598-023-42603-2. 37903815 PMC10616186

[30] C. K. Kusumah , “12‐Years Compulsory Education Policy and Education Participation Completeness: Evidence From Indonesia,” Journal of Indonesian Sustainable Development Planning 2, no. 2 (2021): 187–201, 10.46456/jisdep.v2i2.138.

[31] C. M. A. Santoso , T. Bramantoro , M. C. Nguyen , Z. Bagoly , and A. Nagy , “Factors Affecting Dental Service Utilisation in Indonesia: A Population‐Based Multilevel Analysis,” International Journal of Environmental Research and Public Health 17, no. 15 (2020): 5282, 10.3390/ijerph17155282. 32707974 PMC7432444

[32] V. Wiseman , H. Thabrany , A. Asante , et al., “An Evaluation of Health Systems Equity in Indonesia: Study Protocol,” International Journal for Equity in Health 17 (2018): 138, 10.1186/s12939-018-0822-0. 30208921 PMC6134712

[33] U. Kohler , K. B. Karlson , and A. Holm , “Comparing Coefficients of Nested Nonlinear Probability Models,” Stata Journal 11, no. 3 (2011): 420–438, 10.1177/1536867X1101100306.

[34] S. Kiuchi , U. Cooray , T. Kusama , et al., “Oral Status and Dementia Onset: Mediation of Nutritional and Social Factors,” Journal of Dental Research 101, no. 4 (2022): 420–427, 10.1177/00220345211049399. 34796750

[35] A. Ghanbarzadegan , M. Mittinty , D. S. Brennan , and L. M. Jamieson , “The Effect of Education on Dental Service Utilization Patterns in Different Sectors: A Multiple Mediation Analysis,” Community Dentistry and Oral Epidemiology 51, no. 6 (2023): 1093–1099, 10.1111/cdoe.12838. 36576011 PMC10946604

[36] I. Dewanto , S. Koontongkaew , and N. Widyastuti , “Barriers to the Implementation of Dental Insurance in Indonesia as Perceived by Primary Dentists,” Journal of Indonesian Dental Association 1, no. 1 (2018): 281, 10.32793/jida.v1i1.281.

[37] S. Khairinisa , F. Setiawati , R. R. Darwita , and D. A. Maharani , “Perceived Barriers Among Indonesian General Dentists in Providing Caries Preventive Care for Pediatric Patients,” European Journal of Dentistry 18 (2023): 632–639, 10.1055/s-0043-1771336. 37591284 PMC11132772

[38] The Lancet Public Health , “Education: A Neglected Social Determinant of Health,” Lancet Public Health 5, no. 7 (2020): e361, 10.1016/S2468-2667(20)30144-4. 32619534 PMC7326385

[39] J. Y. Lee , “Economic Inequality, Social Determinants of Health, and the Right to Social Security,” Health and Human Rights 25, no. 2 (2023): 155–169. 38145137 PMC10733760

[40] M. Abdelrehim and S. Singhal , “Is Private Insurance Enough to Address Barriers to Accessing Dental Care? Findings From a Canadian Population‐Based Study,” BMC Oral Health 24 (2024): 503, 10.1186/s12903-024-04271-0. 38685013 PMC11057150

[41] H. Leggett , J. Csikar , K. Vinall‐Collier , and G. V. A. Douglas , “Whose Responsibility Is It Anyway? Exploring Barriers to Prevention of Oral Diseases Across Europe,” JDR Clinical & Translational Research 6, no. 1 (2021): 96–108, 10.1177/2380084420926972. 32437634 PMC7754828

[42] A. D. Laksono , R. Rukmini , and R. D. Wulandari , “Regional Disparities in Antenatal Care Utilization in Indonesia,” PLoS One 15, no. 2 (2020): e0224006, 10.1371/journal.pone.0224006. 32053621 PMC7018075

[43] C. Pardo and W. Schott , “Public Versus Private: Evidence on Health Insurance Selection,” International Journal of Health Care Finance and Economics 12, no. 1 (2012): 39–61, 10.1007/s10754-012-9105-2. 22374192 PMC3367436

[44] I. A. Odeyemi and J. Nixon , “The Role and Uptake of Private Health Insurance in Different Health Care Systems: Are There Lessons for Developing Countries?,” Clinical Outcomes Research CEOR 5 (2013): 109–118, 10.2147/CEOR.S40386. PMC3593711 23494071

[45] Y. Watanabe , “Long‐Run Measurement of Income‐Related Inequalities in Health Care Under Universal Coverage: Evidence From Longitudinal Analysis in Korea,” Health Economics Review 14 (2024): 86, 10.1186/s13561-024-00557-9. 39387941 PMC11465630

[46] Oxford Academic , “Measuring Progress Towards Universal Health Coverage: With an Application to 24 Developing Countries,” Oxford Review of Economic Policy, accessed May 22, 2024, https://academic.oup.com/oxrep/article/32/1/147/2452826.

[47] T. Boerma , P. Eozenou , D. Evans , T. Evans , M. P. Kieny , and A. Wagstaff , “Monitoring Progress Towards Universal Health Coverage at Country and Global Levels,” PLoS Medicine 11, no. 9 (2014): e1001731, 10.1371/journal.pmed.1001731. 25243899 PMC4171369

[48] R. G. Watt , M. R. Mathur , J. Aida , M. Bönecker , R. Venturelli , and S. A. Gansky , “Oral Health Disparities in Children: A Canary in the Coalmine?,” Pediatric Clinics of North America 65, no. 5 (2018): 965–979, 10.1016/j.pcl.2018.05.006. 30213357

[49] P. A. Braveman , S. Kumanyika , J. Fielding , et al., “Health Disparities and Health Equity: The Issue Is Justice,” American Journal of Public Health 101, no. Suppl. 1 (2011): S149–S155, 10.2105/AJPH.2010.300062. 21551385 PMC3222512

[50] A. Wagstaff , H. T. H. Nguyen , H. Dao , and S. Bales , “Encouraging Health Insurance for the Informal Sector: A Cluster Randomized Experiment in Vietnam,” Health Economics 25, no. 6 (2016): 663–674, 10.1002/hec.3293. 26666771

[51] Q. Cheng , A. Asante , D. Susilo , et al., “Equity of Health Financing in Indonesia: A 5‐Year Financing Incidence Analysis (2015–2019),” Lancet Regional Health – Western Pacific 21 (2022): 100400, 10.1016/j.lanwpc.2022.100400. 35243456 PMC8873956

