# Supplementary material for: Mediating Role of Health Insurance on Socioeconomic Inequalities in Dental Utilisation Patterns Among Indonesian Adults
Source: Community Dent Oral Epidemiol. 2025 Aug 24;53(6):685–95. doi: 10.1111/cdoe.70013 (PMC12627263; doi:10.1111/cdoe.70013)
Supplement: Supplementary file 1 — Tables S1–S2: cdoe70013‐sup‐0001‐TableS1‐S2.docx. [file CDOE-53-685-s001.docx]

**Supplementary Material 1 :** Sensitivity Analysis for KHB Mediation Analysis

Table S1.1 Odd Ratios of Economic Status and Health Insurance for Dental Utilization by Multinomial Logistic Regression (n=26,351)

|  | Model 1^a^ | | | | Model 2^b^ | | | | Model 3^c^ | | | |
| --- | --- | --- | --- | --- | --- | --- | --- | --- | --- | --- | --- | --- |
|  | OR | 95% CI | | p-value | OR | 95% CI | | p-value | OR | 95% CI | | p-value |
|  |  | Lower | Upper |  |  | Lower | Upper |  |  | Lower | Upper |  |
| **Irregular User** |  |  |  |  |  |  |  |  |  |  |  |  |
| **Public Insurance (Ref: No)** |  |  |  |  |  |  |  |  |  |  |  |  |
| Yes |  |  |  |  |  |  |  |  | 1.42 | 1.31 | 1.53 | **<0.001** |
| **Private Insurance (Ref: No)** |  |  |  |  |  |  |  |  |  |  |  |  |
| Yes |  |  |  |  |  |  |  |  | 2.08 | 1.85 | 2.34 | **<0.001** |
| **Economic Status (Ref: Q1)** |  |  |  |  |  |  |  |  |  |  |  |  |
| Q2 | 1.30 | 1.13 | 1.50 | **<0.001** | 1.23 | 1.06 | 1.42 | **0.01** | 1.23 | 1.06 | 1.42 | **0.01** |
| Q3 | 1.64 | 1.43 | 1.88 | **<0.001** | 1.54 | 1.34 | 1.77 | **<0.001** | 1.52 | 1.32 | 1.74 | **<0.001** |
| Q4 | 2.41 | 2.12 | 2.75 | **<0.001** | 2.23 | 1.95 | 2.55 | **<0.001** | 2.14 | 1.87 | 2.45 | **<0.001** |
| Q5 (High) | 3.94 | 3.48 | 4.47 | **<0.001** | 3.53 | 3.11 | 4.01 | **<0.001** | 3.22 | 2.83 | 3.67 | **<0.001** |
| **Regular User** |  |  |  |  |  |  |  |  |  |  |  |  |
| **Public Insurance (Ref: No)** |  |  |  |  |  |  |  |  |  |  |  |  |
| Yes |  |  |  |  |  |  |  |  | 1.45 | 1.17 | 1.80 | **<0.001** |
| **Private Insurance (Ref: No)** |  |  |  |  |  |  |  |  |  |  |  |  |
| Yes |  |  |  |  |  |  |  |  | 6.82 | 5.41 | 8.60 | **<0.001** |
| **Economic Status (Ref: Q1)** |  |  |  |  |  |  |  |  |  |  |  |  |
| Q2 | 2.35 | 1.31 | 4.23 | **<0.001** | 2.08 | 1.16 | 3.76 | 0.02 | 1.99 | 1.10 | 3.60 | 0.02 |
| Q3 | 3.16 | 1.79 | 5.56 | **<0.001** | 2.76 | 1.56 | 4.87 | **<0.001** | 2.42 | 1.37 | 4.28 | **<0.001** |
| Q4 | 5.73 | 3.35 | 9.79 | **<0.001** | 4.83 | 2.82 | 8.29 | **<0.001** | 3.88 | 2.25 | 6.70 | **<0.001** |
| Q5 (High) | 13.45 | 8.05 | 22.47 | **<0.001** | 10.87 | 6.47 | 18.25 | **<0.001** | 7.23 | 4.27 | 12.25 | **<0.001** |

OR: Odd Ratio, CI: Confidence Interval

^a^Exposure (Economic Status)

^b^Exposure (Economic Status) + Confounders (sex, age, ethnicity, religion, marital status, household size, and residency)

^c^Exposure (Economic Status) + Mediator (Public Insurance and Private Insurance) + Confounders

Table S1.2 Odd Ratios of Education Status and Health Insurance for Dental Utilization by Multinomial Logistic Regression (n=26,351)

|  | Model 1^a^ | | | | Model 2^b^ | | | | Model 3^c^ | | | |
| --- | --- | --- | --- | --- | --- | --- | --- | --- | --- | --- | --- | --- |
|  | OR | 95% CI | | p-value | OR | 95% CI | | p-value | OR | 95% CI | | p-value |
|  |  | Lower | Upper |  |  | Lower | Upper |  |  | Lower | Upper |  |
| **Irregular User** |  |  |  |  |  |  |  |  |  |  |  |  |
| **Public Insurance (Ref: No)** |  |  |  |  |  |  |  |  |  |  |  |  |
| Yes |  |  |  |  |  |  |  |  | 1.29 | 1.19 | 1.39 | **<0.001** |
| **Private Insurance (Ref: No)** |  |  |  |  |  |  |  |  |  |  |  |  |
| Yes |  |  |  |  |  |  |  |  | 1.96 | 1.74 | 2.20 | **<0.001** |
| **Highest Educational Attainment  (Ref: Unschooled)** |  |  |  |  |  |  |  |  |  |  |  |  |
| Elementary school | 1.71 | 1.29 | 2.28 | **<0.001** | 1.91 | 1.43 | 2.55 | **<0.001** | 1.90 | 1.42 | 2.54 | **0.01** |
| Junior High School | 2.27 | 1.70 | 3.03 | **<0.001** | 2.80 | 2.07 | 3.78 | **<0.001** | 2.72 | 2.02 | 3.67 | **<0.001** |
| Senior High School | 4.04 | 3.06 | 5.35 | **<0.001** | 4.69 | 3.49 | 6.29 | **<0.001** | 4.35 | 3.24 | 5.84 | **<0.001** |
| Higher Education | 9.04 | 6.82 | 11.99 | **<0.001** | 10.38 | 7.73 | 13.94 | **<0.001** | 9.12 | 6.79 | 12.27 | **<0.001** |
| **Regular User** |  |  |  |  |  |  |  |  |  |  |  |  |
| **Public Insurance (Ref: No)** |  |  |  |  |  |  |  |  |  |  |  |  |
| Yes |  |  |  |  |  |  |  |  | 1.32 | 1.06 | 1.64 | **<0.001** |
| **Private Insurance (Ref: No)** |  |  |  |  |  |  |  |  |  |  |  |  |
| Yes |  |  |  |  |  |  |  |  | 6.44 | 5.11 | 8.12 | **<0.001** |
| **Highest Educational Attainment  (Ref: Unschooled)** |  |  |  |  |  |  |  |  |  |  |  |  |
| Unschooled |  |  |  |  |  |  |  |  |  |  |  |  |
| Elementary school | 1.67 | 0.51 | 5.45 | 0.39 | 1.45 | 0.44 | 4.78 | 0.54 | 1.49 | 0.45 | 4.91 | 0.51 |
| Junior High School | 2.20 | 0.66 | 7.30 | 0.20 | 1.78 | 0.53 | 6.05 | 0.35 | 1.69 | 0.49 | 5.74 | 0.40 |
| Senior High School | 8.59 | 2.73 | 27.02 | **<0.001** | 6.03 | 1.86 | 19.50 | **<0.001** | 4.72 | 1.45 | 15.35 | **0.01** |
| Higher Education | 24.89 | 7.93 | 78.12 | **<0.001** | 17.27 | 5.35 | 55.71 | **<0.001** | 11.50 | 3.54 | 37.39 | **<0.001** |

OR: Odd Ratio, CI: Confidence Interval

^a^Exposure (Education Status)

^b^Exposure (Education Status) + Confounders (sex, age, ethnicity, religion, marital status, household size, and residency)

^c^Exposure (Education Status) + Mediator (Public Insurance and Private Insurance) + Confounder

**Supplementary Material 2 :** KHB Mediation Analysis for each Public and Private Insurance Ownership

Table S2.1 Mediation analysis between Economic and Educational Status with Dental Utilization through Public Insurance (n=26,351)

|  | **Irregular Utilization** | | | | | | **Regular Utilization** | | | | | |
| --- | --- | --- | --- | --- | --- | --- | --- | --- | --- | --- | --- | --- |
|  | Model 1 | | | Model 2 | | | Model 1 | | | Model 2 | | |
|  | OR | 95%CI Lower | 95%CI Upper | OR | 95%CI Lower | 95%CI Upper | OR | 95%CI Lower | 95%CI Upper | OR | 95%CI Lower | 95%CI Upper |
| Economic Status (Ref: Q1) |  |  |  |  |  |  |  |  |  |  |  |  |
| **Q2** |  |  |  |  |  |  |  |  |  |  |  |  |
| Total Effect | 1.30** | 1.1 | 1.50 | 1.23** | 1.06 | 1.42 | 2.36** | 1.31 | 4.24 | 2.08** | 1.16 | 3.76 |
| Direct Effect | 1.31** | 1.13 | 1.51 | 1.24** | 1.07 | 1.43 | 2.37** | 1.32 | 4.27 | 2.11** | 1.17 | 3.80 |
| Indirect Effect | 0.99 | 0.98 | 1.01 | 0.99 | 0.98 | 1.01 | 0.99 | 0.97 | 1.01 | 0.99 | 0.97 | 1.01 |
| %PM by Public Insurance | -2.2 |  |  | -4.3 |  |  | -0.8 |  |  | -1.4 |  |  |
| **Q3** |  |  |  |  |  |  |  |  |  |  |  |  |
| Total Effect | 1.64** | 1.43 | 1.88 | 1.54** | 1.34 | 1.77 | 3.17** | 1.80 | 5.58 | 2.75** | 1.56 | 4.86 |
| Direct Effect | 1.66** | 1.44 | 1.90 | 1.56** | 1.36 | 1.79 | 3.21** | 1.82 | 5.65 | 2.80** | 1.59 | 4.95 |
| Indirect Effect | 0.99 | 0.97 | 1.01 | 0.99 | 0.97 | 1.00 | 0.99 | 0.97 | 1.01 | 0.98 | 0.96 | 1.00 |
| %PM by Public Insurance | -2.3 |  |  | -3.4 |  |  | -1.2 |  |  | -1.7 |  |  |
| **Q4** |  |  |  |  |  |  |  |  |  |  |  |  |
| Total Effect | 2.42** | 2.13 | 2.76 | 2.24** | 1.96 | 2.55 | 5.76** | 3.36 | 9.85 | 4.84** | 2.82 | 8.32 |
| Direct Effect | 2.42** | 2.12 | 2.76 | 2.25** | 1.97 | 2.57 | 5.74** | 3.35 | 9.82 | 4.88** | 2.84 | 8.37 |
| Indirect Effect | 1.00 | 0.99 | 1.02 | 0.99 | 0.98 | 1.01 | 1.00 | 0.98 | 1.02 | 0.99 | 0.98 | 1.01 |
| %PM by Public Insurance | 0.3 |  |  | -0.7 |  |  | 0.2 |  |  | -0.4 |  |  |
| **Q5** |  |  |  |  |  |  |  |  |  |  |  |  |
| Total Effect | 3.98** | 3.51 | 4.51 | 3.55** | 3.12 | 4.03 | 13.59** | 8.13 | 22.70 | 10.94** | 6.51 | 18.37 |
| Direct Effect | 3.93** | 3.47 | 4.45 | 3.55** | 3.12 | 4.03 | 13.37** | 8.01 | 22.34 | 10.93** | 6.51 | 18.35 |
| Indirect Effect | 1.01 | 1.00 | 1.03 | 1.00 | 0.99 | 1.01 | 1.02 | 1.00 | 1.04 | 1.00 | 0.98 | 1.02 |
| %PM by Public Insurance | 1.0 |  |  | 0.1 |  |  | 0.6 |  |  | 0.0 |  |  |
| Education Status (Ref: Unschooled) | | | |  |  |  |  |  |  |  |  |  |
| **Elementary School** |  |  |  |  |  |  |  |  |  |  |  |  |
| Total Effect | 1.71** | 1.29 | 2.28 | 1.91** | 1.43 | 2.55 | 1.67** | 0.51 | 5.45 | 1.43** | 0.44 | 4.73 |
| Direct Effect | 1.69** | 1.27 | 2.25 | 1.88** | 1.41 | 2.52 | 1.65** | 0.51 | 5.38 | 1.42** | 0.43 | 4.66 |
| Indirect Effect | 1.01 | 0.99 | 1.03 | 1.01 | 0.99 | 1.03 | 1.01 | 0.99 | 1.04 | 1.01 | 0.99 | 1.04 |
| %PM by Public Insurance | 2.5 |  |  | 1.8 |  |  | 2.8 |  |  | 3.8 |  |  |
| **Middle School** |  |  |  |  |  |  |  |  |  |  |  |  |
| Total Effect | 2.28** | 1.70 | 3.04 | 2.78** | 2.06 | 3.75 | 2.20** | 0.66 | 7.31 | 1.77** | 0.52 | 5.99 |
| Direct Effect | 2.24** | 1.68 | 3.00 | 2.74** | 2.03 | 3.69 | 2.17** | 0.65 | 7.20 | 1.73** | 0.51 | 5.89 |
| Indirect Effect | 1.01 | 0.99 | 1.03 | 1.02 | 1.00 | 1.03 | 1.01 | 0.99 | 1.04 | 1.02 | 0.99 | 1.04 |
| %PM by Public Insurance | 1.7 |  |  | 1.5 |  |  | 1.8 |  |  | 3.2 |  |  |
| **High School** |  |  |  |  |  |  |  |  |  |  |  |  |
| Total Effect | 4.06** | 3.06 | 5.37 | 4.65** | 3.47 | 6.24 | 8.62** | 2.74 | 27.11 | 6.11** | 1.89 | 19.75 |
| Direct Effect | 3.93** | 2.97 | 5.20 | 4.55** | 3.39 | 6.10 | 8.35** | 2.65 | 26.25 | 5.93** | 1.83 | 19.20 |
| Indirect Effect | 1.03* | 1.01 | 1.05 | 1.02* | 1.01 | 1.04 | 1.03* | 1.00 | 1.06 | 1.03* | 1.00 | 1.06 |
| %PM by Public Insurance | 2.2 |  |  | 1.5 |  |  | 1.5 |  |  | 1.6 |  |  |
| **Higher Education** |  |  |  |  |  |  |  |  |  |  |  |  |
| Total Effect | 9.11** | 6.87 | 12.08 | 10.33** | 7.69 | 13.88 | 25.08** | 7.99 | 78.74 | 17.80** | 5.52 | 57.37 |
| Direct Effect | 8.56** | 6.46 | 11.36 | 9.86** | 7.34 | 13.24 | 23.52** | 7.49 | 73.87 | 16.80** | 5.21 | 54.21 |
| Indirect Effect | 1.06* | 1.04 | 1.09 | 1.05* | 1.03 | 1.07 | 1.07* | 1.02 | 1.11 | 1.06* | 1.01 | 1.11 |
| %PM by Public Insurance | 2.8 |  |  | 2.0 |  |  | 2.0 |  |  | 2.0 |  |  |

OR: Odd Ratio, CI: Confidence Interval, PM: Proportion Mediated

Model 1 = Unadjusted model (Economic ***or*** Education status 🡪 Public Insurance 🡪 Dental Utilization)

Model 2=Model 1 adjusted with confounders (sex, age, ethnicity, religion, marital status, household size, and residency)

*Significant at p < 0.05; **Significant at p < 0.01

Table S2.2 Mediation analysis between Economic and Educational Status with Dental Utilization through Private Insurance (n=26,351)

|  | **Irregular Utilization** | | | | | | **Regular Utilization** | | | | | |
| --- | --- | --- | --- | --- | --- | --- | --- | --- | --- | --- | --- | --- |
|  | Model 1 | | | Model 2 | | | Model 1 | | | Model 2 | | |
|  | OR | 95%CI Lower | 95%CI Upper | OR | 95%CI Lower | 95%CI Upper | OR | 95%CI Lower | 95%CI Upper | OR | 95%CI Lower | 95%CI Upper |
| Economic Status (Ref: Q1) |  |  |  |  |  |  |  |  |  |  |  |  |
| **Q2** |  |  |  |  |  |  |  |  |  |  |  |  |
| Total Effect | 1.29** | 1.12 | 1.49 | 1.23** | 1.06 | 1.42 | 2.20** | 1.22 | 3.96 | 2.02** | 1.12 | 3.65 |
| Direct Effect | 1.27** | 1.10 | 1.47 | 1.22** | 1.05 | 1.41 | 2.11** | 1.17 | 3.80 | 1.97** | 1.09 | 3.57 |
| Indirect Effect | 1.02* | 1.00 | 1.03 | 1.01* | 1.00 | 1.02 | 1.04** | 1.01 | 1.08 | 1.02 | 0.99 | 1.06 |
| %PM by Private Insurance | 6.7 |  |  | 4.4 |  |  | 5.5 |  |  | 3.3 |  |  |
| **Q3** |  |  |  |  |  |  |  |  |  |  |  |  |
| Total Effect | 1.62** | 1.41 | 1.86 | 1.53** | 1.33 | 1.76 | 2.81** | 1.59 | 4.95 | 2.54** | 1.43 | 4.49 |
| Direct Effect | 1.57** | 1.37 | 1.80 | 1.50** | 1.30 | 1.72 | 2.58** | 1.46 | 4.55 | 2.40** | 1.35 | 4.24 |
| Indirect Effect | 1.03** | 1.02 | 1.05 | 1.02* | 1.01 | 1.04 | 1.09** | 1.05 | 1.13 | 1.06** | 1.03 | 1.09 |
| %PM by Private Insurance | 7.0 |  |  | 5.2 |  |  | 8.3 |  |  | 6.2 |  |  |
| **Q4** |  |  |  |  |  |  |  |  |  |  |  |  |
| Total Effect | 2.39** | 2.10 | 2.72 | 2.22** | 1.94 | 2.53 | 4.83** | 2.81 | 8.28 | 4.31** | 2.50 | 7.42 |
| Direct Effect | 2.25** | 1.97 | 2.57 | 2.12** | 1.85 | 2.42 | 4.14** | 2.41 | 7.12 | 3.84** | 2.23 | 6.63 |
| Indirect Effect | 1.06* | 1.05 | 1.08 | 1.05** | 1.03 | 1.06 | 1.17** | 1.12 | 1.21 | 1.12** | 1.08 | 1.16 |
| %PM by Private Insurance | 6.9 |  |  | 5.5 |  |  | 9.7 |  |  | 7.8 |  |  |
| **Q5** |  |  |  |  |  |  |  |  |  |  |  |  |
| Total Effect | 3.92** | 3.46 | 4.44 | 3.51** | 3.09 | 3.99 | 10.74** | 6.40 | 18.01 | 9.04** | 5.35 | 15.27 |
| Direct Effect | 3.49** | 3.07 | 3.96 | 3.20** | 2.81 | 3.64 | 7.98** | 4.74 | 13.45 | 7.12** | 4.20 | 12.07 |
| Indirect Effect | 1.12** | 1.10 | 1.15 | 1.10** | 1.08 | 1.12 | 1.35** | 1.28 | 1.41 | 1.27** | 1.22 | 1.32 |
| %PM by Private Insurance | 8.5 |  |  | 7.4 |  |  | 12.5 |  |  | 10.8 |  |  |
| Education Status (Ref: Unschooled) | | | |  |  |  |  |  |  |  |  |  |
| **Elementary School** |  |  |  |  |  |  |  |  |  |  |  |  |
| Total Effect | 1.71** | 1.29 | 2.27 | 1.92** | 1.44 | 2.57 | 1.60** | 0.49 | 5.21 | 1.50** | 0.45 | 4.94 |
| Direct Effect | 1.69** | 1.27 | 2.25 | 1.93** | 1.44 | 2.57 | 1.56** | 0.48 | 5.07 | 1.51** | 0.46 | 4.97 |
| Indirect Effect | 1.01* | 1.00 | 1.02 | 1.00 | 0.99 | 1.01 | 1.03** | 1.00 | 1.06 | 0.99 | 0.96 | 1.03 |
| %PM by Private Insurance | 1.8 |  |  | -0.4 |  |  | 5.7 |  |  | -1.9 |  |  |
| **Middle School** |  |  |  |  |  |  |  |  |  |  |  |  |
| Total Effect | 2.25** | 1.69 | 3.01 | 2.81** | 2.08 | 3.80 | 1.94** | 0.58 | 6.45 | 1.78** | 0.52 | 6.07 |
| Direct Effect | 2.19** | 1.64 | 2.92 | 2.79** | 2.06 | 3.76 | 1.79** | 0.54 | 5.93 | 1.73** | 0.51 | 5.91 |
| Indirect Effect | 1.03** | 1.02 | 1.04 | 1.01 | 1.00 | 1.02 | 1.09** | 1.05 | 1.12 | 1.03** | 1.00 | 1.06 |
| %PM by Private Insurance | 3.7 |  |  | 1.0 |  |  | 12.6 |  |  | 4.9 |  |  |
| **High School** |  |  |  |  |  |  |  |  |  |  |  |  |
| Total Effect | 3.99** | 3.02 | 5.29 | 4.70** | 3.51 | 6.31 | 6.88** | 2.18 | 21.69 | 5.57** | 1.71 | 18.13 |
| Direct Effect | 3.70** | 2.80 | 4.90 | 4.48** | 3.34 | 6.02 | 5.58** | 1.77 | 17.60 | 4.88** | 1.50 | 15.88 |
| Indirect Effect | 1.08** | 1.06 | 1.10 | 1.05 | 1.03 | 1.06 | 1.23** | 1.19 | 1.28 | 1.14** | 1.10 | 1.18 |
| %PM by Private Insurance | 5.4 |  |  | 3.1 |  |  | 10.9 |  |  | 7.7 |  |  |
| **Higher Education** |  |  |  |  |  |  |  |  |  |  |  |  |
| Total Effect | 9.03** | 6.81 | 11.98 | 10.51** | 7.82 | 14.12 | 19.74** | 6.27 | 62.10 | 15.49** | 4.77 | 50.33 |
| Direct Effect | 8.01** | 6.04 | 10.63 | 9.58** | 7.13 | 12.88 | 14.11** | 4.47 | 44.54 | 12.02** | 3.70 | 39.10 |
| Indirect Effect | 1.13** | 1.10 | 1.15 | 1.10** | 1.07 | 1.12 | 1.40** | 1.33 | 1.47 | 1.29** | 1.23 | 1.35 |
| %PM by Private Insurance | 5.5 |  |  | 3.9 |  |  | 11.2 |  |  | 9.3 |  |  |

OR: Odd Ratio, CI: Confidence Interval, PM: Proportion Mediated

Model 1 = Unadjusted model (Economic ***or*** Education status 🡪 Private Insurance 🡪 Dental Utilization)

Model 2=Model 1 adjusted with confounders (sex, age, ethnicity, religion, marital status, household size, and residency)

*Significant at p < 0.05; **Significant at p < 0.01
